# Failure of fluconazole in treating cutaneous leishmaniasis caused by *Leishmania guyanensis* in the Brazilian Amazon: An open, nonrandomized phase 2 trial

**DOI:** 10.1371/journal.pntd.0006225

**Published:** 2018-02-26

**Authors:** Valeska Albuquerque Francesconi, Fabio Francesconi, Rajendranath Ramasawmy, Gustavo Adolfo Sierra Romero, Maria das Graças Costa Alecrim

**Affiliations:** 1 Department of Dermatology, Academic and Research Division at the Tropical Medicine Foundation - Dr Heitor Vieira Dourado, Manaus, Amazonas, Brazil; 2 Department of Molecular Biology, Division of Immunogenetics, at the Tropical Medicine Foundation - Dr Heitor Vieira Dourado and Universidade Nilton Lins, Manaus, Amazonas, Brazil; 3 Center for Tropical Medicine, Faculty of Medicine, University of Brasília, Brasília, Federal District, Brazil; 4 Department of Infectology, Division of Malaria and Neglected Tropical Diseases at the Tropical Medicine Foundation - Dr Heitor Vieira Dourado, Manaus, Amazonas, Brazil; National Institute of Allergy and Infectious Diseases, UNITED STATES

## Abstract

**Background:**

The treatment of Leishmaniasis caused by *Leishmania (Viannia) guyanensis* is based on a weak strength of evidence from very few clinical trials and some case series reports. Current treatment guidelines recommend pentamidine isethionate or meglumine antimoniate (Glucantime) as the first-line choices. Both are parenteral drugs with a low therapeutic indexes leading to a high risk of undesired effects. Imidazole derivatives interfere with the production of leishmanial ergosterol, an essential component of their membrane structure. One drug that has been studied in different clinical presentations of *Leishmania* is fluconazole, a hydrophilic bis-triazole, which is easily absorbed through the oral route with a low toxicity profile and is considered safe for children. This drug is readily available in poor countries with a reasonable cost making it a potential option for treating leishmaniasis.

**Methods and findings:**

An adaptive nonrandomized clinical trial with sequential groups with dose escalation of oral fluconazole was designed to treat adult men with localized cutaneous leishmaniasis (LCL) in Manaus, Brazil. Eligible participants were patients with LCL with confirmed *Leishmania guyanensis* infection.

**Results:**

Twenty adult male patients were treated with 450 mg of fluconazole daily for 30 days. One patient (5%) was cured within 30 days of treatment. Of the 19 failures (95%), 13 developed a worsening of ulcers and six evolved lymphatic spreading of the disease. Planned dose escalation was suspended after the disappointing failure rate during the first stage of the trial.

**Conclusion/Significance:**

Oral fluconazole, at the dose of 450mg per day, was not efficacious against LCL caused by *Leishmania guyanensis* in adult men.

**Trial registration:**

Brazilian Clinical Trial Registration (ReBec)—RBR-8w292w; UTN number—1158-2421

## Author summary

The main agent causing localized cutaneous leishmaniasis in the Guianan Ecoregion Complex, a region that covers Guiana, Suriname, French Guiana, the southern portion of Venezuela and the northern portion of the Amazon Basin, is *Leishmania (Viannia) guyanensis*. The current treatment regimens for treating this parasite follow a weak strength of recommendation based on a low-quality evidence. All the drugs used are parenteral drugs with low therapeutic indexes and inadequate cure rates. Azoles are drugs that interfere with the production of leishmanial ergosterol and have been studied in other *Leishmania* species with promising results. Fluconazole is a hydrophilic bis-triazole that accumulates rapidly and extensively in the skin with a low toxicity profile. It is considered safe for children. Oral fluconazole is readily available and has a reasonable cost. In this work, we estimated the efficacy of fluconazole in treating localized cutaneous leishmaniasis caused by *L*. *guyanensis* in Manaus. The study had to be stopped due to the disappointing results, with only a 5% cure rate. Among the failures, 55% developed a marked worsening of their ulcers and 55% of those patients had lymphatic dissemination of the infection. In conclusion, fluconazole at the dose of 450mg per day is not efficacious in treating localized cutaneous leishmaniasis caused by *L*. *guyanensis*.

## Introduction

Cutaneous leishmaniasis (CL) is one of the 17 neglected diseases, with 350 million people at risk in 98 endemic countries [1]. Latin America is a highly endemic region with a documented 30% increase in the number of reported cases during a period of ten years (2001–2011) [2]. Brazil together with eight other countries reports 90% of all registered cases of CL [1]. The incidence of CL in the Brazilian Amazon region surpasses the Brazilian national average (15.3 cases/100.000 inhabitants), reaching in some regions the incidence of 30 cases/100,000 inhabitants [3].

American tegumentary leishmaniasis (ATL), especially in the Amazon region, has a peculiar epidemiological profile characterized by higher intra- and inter-specific variations and heterogeneity of transmission cycles, reservoir hosts and sand fly vectors, with sympatric circulation of various *Leishmania* species. These characteristics altogether lead to diverse clinical presentations and distinct clinical responses to treatment [4].

*Leishmania guyanensis* is the most common cause of human leishmaniasis in the Guianan Ecoregion Complex (GEC) a region that covers Guiana, Suriname, French Guiana, the southern portion of Venezuela and the northern portion of the Amazon Basin [5, 6]. Cutaneous leishmaniasis is the most common clinical presentation of the infection caused by *Leishmania guyanensis* [7]. Lymphatic involvement, manifested as adenomegaly or lymphangitis, is the second most common presentation of the disease affecting 60% of the cases [8]. Disseminated cutaneous leishmaniasis and mucosal disease are less common presentations of leishmaniasis caused by this *Leishmania* species [9, 10].

The current treatment regimens for CL caused by *Leishmania guyanensis* follow a weak strength of recommendation based on a low-quality evidence [11]. Intramuscular administration of 3 mg/kg pentamidine isethionate every other day for up to four injections is considered the treatment of choice [12]. Meglumine antimoniate (Glucantime) 20mg S^v^/Kg/day for 20 days is the recommended treatment in Brazil with a cure ratebetween 53% and 70% [13, 14]. Miltefosine has been used in some studies with a cure rate between 54% and 72% [15, 16], although that drug is not available in South America. Amphotericin B is the drug choice in severe cases.

The present treatment options are parenteral drugs with low cure rates and low therapeutic indexes leading to a high risk for undesired effects. This scenario leads us to research a new therapeutic option.

One drug that has been studied in different clinical presentations of *Leishmania* is fluconazole. This agent is a hydrophilic bis-triazole that is easily absorbed via the oral route and interferes with the production of leishmanial ergosterol, an essential component of the membrane structure [17]. Fluconazole, has a low toxicity profile, it accumulates rapidly and extensively in the skin, and it is readily available, with a reasonable cost. Fluconazole is also considered safe for children [18, 19].

This adaptive phase II trial evaluated the efficacy of fluconazole in the treatment of cutaneous leishmaniasis caused by *L*. *guyanensis*.

## Methods and material

### Patients and methods

**Fig 1:**
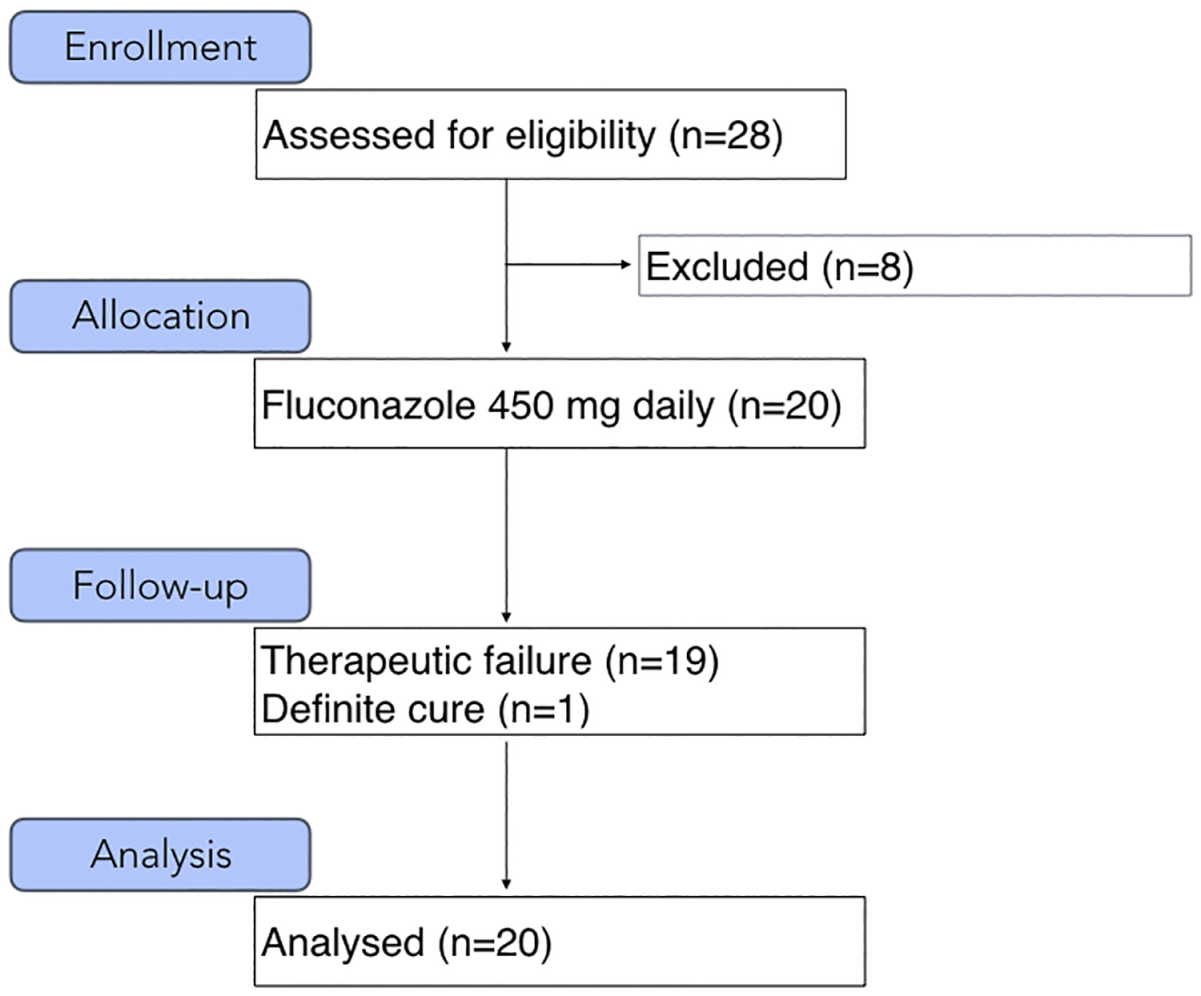
Flow diagram of the progress through the phases: enrolment, intervention allocation, follow-up, and data analysis.

From December 2014 through February 2016, twenty-eight subjects with parasitological confirmed diagnosis of LCL were recruited at the Tropical Medicine Foundation Dr. Heitor Vieira Dourado, in the state of Amazonas, North Brazil, an endemic area of *L*. *guyan*ensis infection. The flow diagram of participants through the different study phases is described in Fig 1.

### Case definition of localized cutaneous leishmaniasis

Localized cutaneous leishmaniasis was defined as the presence of up to five ulcerous lesions without lymphatic or mucousal disease, with amastigotes visualized in direct examination of Giemsa-stained smears of a dermal scrapping taken from the ulcerated border of at least one lesion.

### Inclusion and exclusion criteria

Inclusion criteria were as follows: (1) diagnosis of LCL based on case definition, (2) illness duration <3 months, (3) male sex with age of at least 18 years, (4) 1 to 5 ulcerated lesions, and (5) no previous treatment for leishmaniasis.

Exclusion criteria were as follows: (1) *Leishmania* species could not be identified; (2) infection caused by species other than *L*. *guyanensis*; (3) any uncontrolled active infectious or severe disease, and (4) an allergy to fluconazole.

### Study procedures

Complete blood cell count, tests for the levels of aspartate and alanine aminotransferase, amylase, lipase, urea, creatinine and glucose, and an electrocardiogram were performed in all participants before therapy and at 30, 60, 90 and 180 days after treatment. All patients were subjected to a rapid human immunodeficiency virus test and serology for hepatitis B and C. All the ulcers were measured and photographed. A biopsy of each ulcer was performed and the material was used for a parasite culture and histopathology. *Leishmania* species were identified as described by Marfurt *et al* [20].

Scheduled patient visits were made 30, 60, 90 and 180 days after beginning the treatment. If a patient did not return to follow-up at the specified time, visits were conducted in the patient’s home on the same day or within 7 days of the missed appointment. Patients’ ulcers were measured with a flexible ruler at the initial visit and at each follow-up visit. Standardized digital photographs of the patients’ lesions were obtained at the same time points.

Patients were monitored for adverse events (AEs) and treatment adherence. Patients returned the blister packs of fluconazole to verify compliance. Clinical and laboratory AEs were graded according to the Common Terminology Criteria for Adverse Events of the National Cancer Institute [21].

### Primary and secondary endpoints

Outcome measures followed the protocols published by Olliaro et al [22]. The primary endpoint was a definitive cure six months after the end of treatment. A definitive cure was defined as the complete epithelialization of all lesions without raised borders, infiltrations, inflammation or crusts. The secondary endpoint included an initial cure defined as complete epithelialization of ulcers two months after the end of treatment. If an initial cure was not attained it was considered a therapeutic failure. Any interruption of the treatment was also considered a therapeutic failure.

### Rescue therapy

All patients included in the therapeutic failure group received a rescue therapy of meglumine antimoniate, 20mg Sb^v^/Kg/day for 20 days.

### Intervention

Fluconazole 150 mg capsules conditioned in blisters packs containing 10 capsules were self-administered orally for 30 days. The first dosing schedule involved 450 mg of fluconazole administered once daily, and the second dosing schedule involved 900 mg of fluconazole administered as two daily doses of 450 mg. Both schedules lasted 30 days.

### Study design sample size and statistical analysis

An adaptive phase II trial was adopted based on the successful use of fluconazole in treating *L*. *(Viannia) braziliensis* in a study published by Sousa et. al [23].

The adaptive trial followed FDA recommendations [24].

The sample size of 30 patients in the first step of the study was calculated considering an estimated cure rate of 60%, a precision of +/- 20% and an alpha error of 5%. The patient was instructed to take three capsules (450mg total dose) orally once daily in the morning for 30 days. If 18 patients had reached the primary endpoint with this regimen, then the second step of the study would have begun.

The second step was designed based on the assumption of an improvement in magnitude of response of at least 10% after doubling the dose of fluconazole. Considering a cure rate of 70%, a precision of +/- 10% and an alpha error of 5%, a sample size of 73 patients was calculated. Patients in the second step would have received three 150 mg fluconazol in the morning and three 150 mg fluconazole in the afternoon (900 mg total dose) for 30 days.

All statistical analyses were performed with SPSS 21.0 software for Windows.

### Ethical aspects

This trial was conducted according to the Declaration of Helsinki. Before they were enrolled in the study, written informed consent was obtained from all patients. The study was approved by the Ethics Committee of the Tropical Medicine Foundation Dr Heitor Vieira Dourado, Brazil - registration number 26118613.4.0000.0005. This clinical trial was registered in ReBEC (Brazilian Registry of Clinical Trials) with the identifier RBR-8w292w and is available from http://www.ensaiosclinicos.gov.br/rg/view/2668/ UTN number–1158–2421

## Results

Twenty-eight adult male patients fulfilled the inclusion criteria and were accepted into the study. Their median age was 38.3 years old (range 18–56). Of those, eight (28.6%) were excluded. In three cases the causative species was not *Leishmania guyanensis*, and in the other five, the parasite species could not be identified. Twenty adult male patients (71.4%) remained in the study for further analysis.

Those 20 patients presented 40 lesions, all with less than 3 months of duration. Fifty-five percent (n = 11) presented with one lesion, 15% (n = 3) with two lesions, 15% (n = 3) with three lesions, 5% (n = 1) with four lesions and 10% (n = 2) with five lesions. Concerning the distribution of the lesions, they were predominantly on exposed areas of the body, with 37.5% (n = 15) located on the lower limbs, 37.5% (n = 15) located on the upper limbs and 15% (n = 6) located on the face. Ten percent of the lesions (n = 4) were located on the trunk.

**Fig 2:**
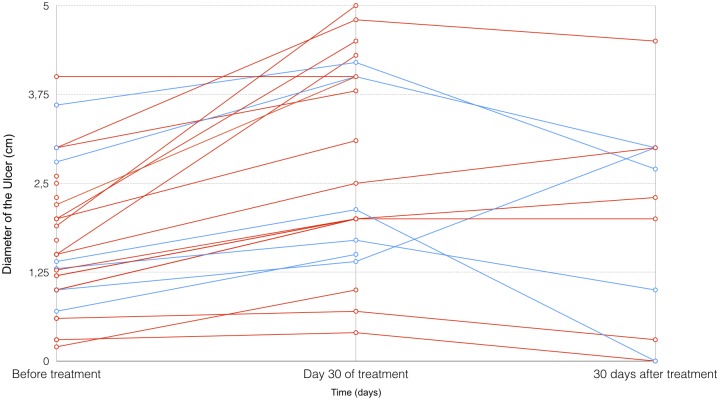
Multi-series line chart representing the size of each ulcer before treatment, at 30 days of treatment and after 30 days after the completion of treatment. The red lines represent increasing ulcer size, and the blue lines represent decreasing ulcer size. The lines that stop at day 30 represent the lesions of patients who suffered treatment failure and requested rescue treatment. Lesions that appeared after 30 days of completing treatment were new lesions.

The median diameter of all 40 ulcers on the first day of treatment was 1.70cm (range 0.2 cm– 4 cm). During the trial, five participants with 17 total ulcers were excluded before the end of 30 days of treatment. At the end of treatment (day 30), 23 lesions on 15 patients remained active ulcers with a median diameter of 2.80 cm (range 0.4 cm– 5 cm). Twelve lesions on six patients remained active ulcers at day 60 (30 days after the end of treatment), with a median diameter of 2.37 cm (range 0.3 cm– 4.5 cm). The clinical evolution of the ulcers is represented in Fig 2.

**Fig 3:**
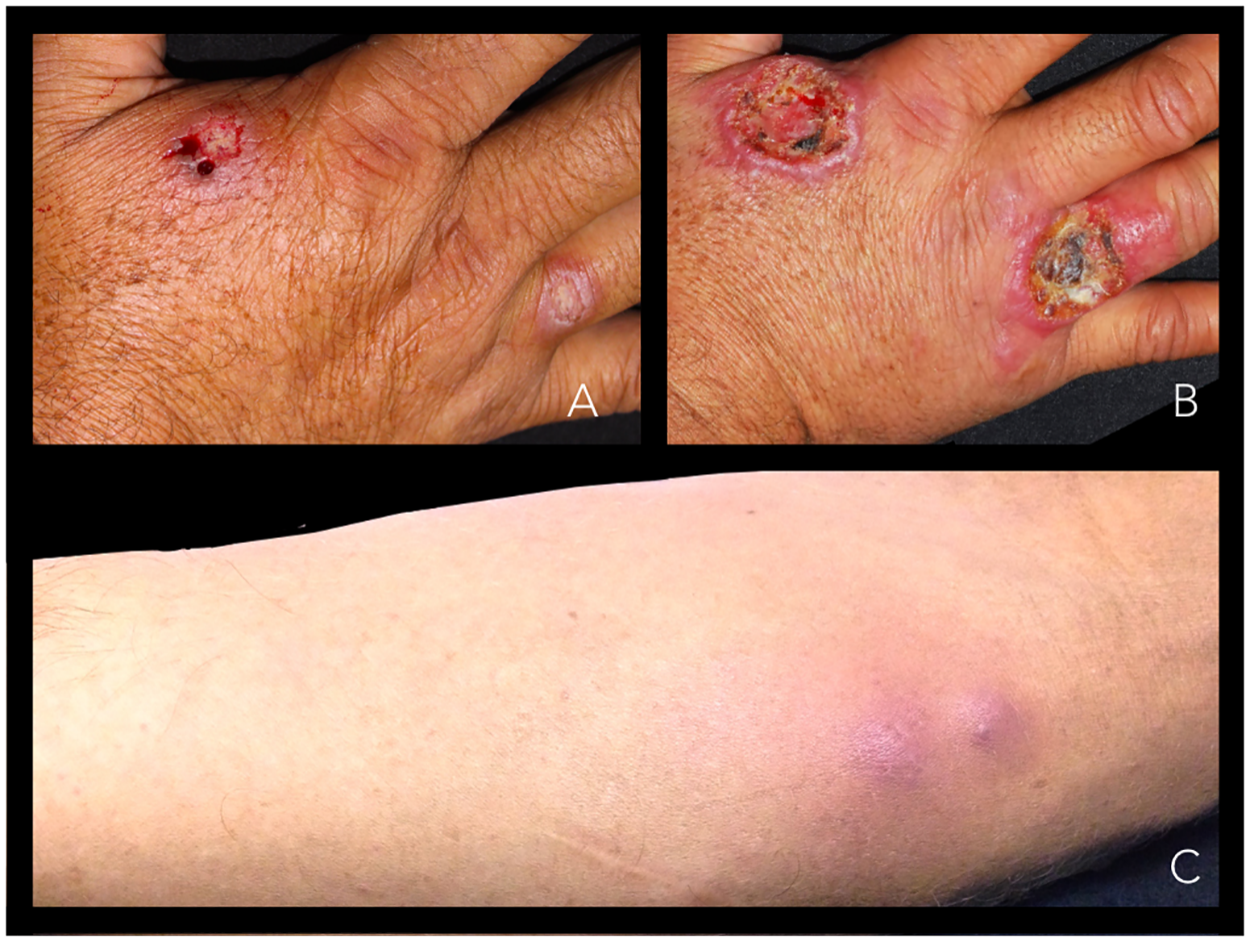
Two examples of clinical worsening with fluconazole 450 mg administered once daily. Picture A shows two lesions on the right hand of the patient before treatment, and picture B shows the same lesions after 30 days of fluconazole. Picture C shows new lesions caused by lymphatic spread in another patient after treatment with fluconazole.

A definitive cure was documented in 5% (n = 1) of the cases, as shown in S1 Fig. The remaining 19 patients were considered treatment failures. Five patients asked to change medication due to ulcer enlargement. In eleven cases (55%) there was worsening of the ulcers with marked inflammatory signs (Fig 3). In six patients (30%) lymphatic spread of the disease was also noted (Fig 3). In those 19 patients, fluconazole treatment was stopped and rescue therapy was instituted with complete resolution of the ulcers.

**Fig 4:**
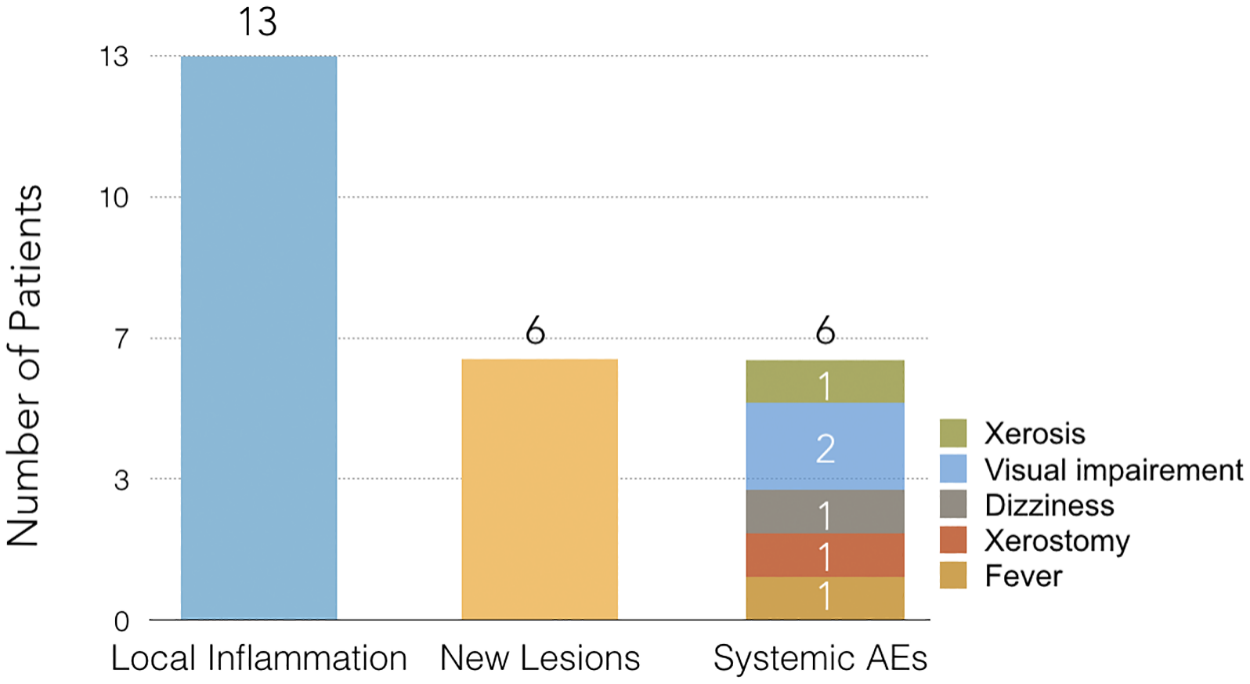
Local inflammatory reactions, all cases of the lymphatic spread of the disease and the adverse events related to fluconazole treatment.

The drug was well tolerated with mild self-limited systemic adverse events in five patients (27.78%) as shown in Fig 4.

## Discussion

The first report of the activity of an azole against a species of the genus *Leishmania* came from an *in vitro* test of CIBA 32,644-Ba in 1965 and another of 2-amino-5-(1-methyl-5-nitro-2-imidazolyl)1-3-thiadiazole in1968 [25, 26]. At the end of the 1960s and in the 1970s, in Brazil, the clinical efficacy of 1-(5-nitro-2-tiazolil) 2-imidazolidinone and niridazole in cutaneous and mucosal leishmaniasis caused by *L*. *(Viannia) braziliensis* was reported, in case series, with some clinical response, at the cost of serious neurologic adverse events [27, 28].

The interest in using azoles in the treatment of leishmaniasis was revived after the report published by Berman indicating the activity of ketoconazole against leishmanial species in macrophage culture [29].

The efficacy of the azoles in treating ATL is mainly based on case series or small trials with heterogeneous results. The cure rate of ketoconazole 400mg bid was as follows: one out of six patients with ATL caused by *L*. *guyanensis* [30], three out of three patients with ATL caused by *L*. *braziliensis* [31] and 16 out of 22 patients with ATL by *L*. *panamensis* [32]. In two studies, itraconazole cured six cases out of ten [33] in one study and three out of 13 patients in the other [34].

The first clinical use of fluconazole in leishmaniasis was against kala-azar with 0% definite cure. Some patients had early apparent cures with later relapsing [35]. In 2002, Alrajhi *et al*. published a randomized, placebo-controlled trial and concluded that a six-week course of 200 mg fluconazole daily was safe and useful to treat CL caused by *L*. *major* [36]. Afterwards, this drug became an alternative for the treatment of Old World cutaneous leishmaniasis. A trial published by Emad *et al*. evidenced that 400mg of fluconazole daily was more efficacious in infections caused by *L*. *major* when compared to 200mg daily, with six-week cure rates of 81% versus 48.3% respectively [37].

In Brazil, a case series was conducted where 28 patients with confirmed leishmaniasis caused by *L*. *(V*.*) braziliensis*, who refused or could not use antimonials, received oral fluconazole for 20 days. Eight patients received 5mg/Kg/day with a cure rate of 75%, 14 patients received 6.5mg/Kg/day with a cure rate of 92.8% and six patients received 8mg/Kg/day with a cure rate of 100% [23]. The authors concluded that there was a higher efficacy at higher doses of fluconazole.

Treatment of leishmaniasis caused by *L*. *guyanensis* follows a weak strength of recommendation based on a low-quality of evidence [11]. Eight published trials analyzed the cure rate in cases of ATL caused by this species, with results varying from 53.6% to 91.7% (S1 Table).

Considering the lack of an optimal treatment for ATL caused by *L*. *guyanensis*, fluconazole seemed to be a promising drug alternative for treating this disease. This assumption was based on the mechanism of action of the drug in the protozoan ergosterol metabolism and on the clinical efficacy evidenced in clinical trials of Old World leishmaniasis and the clinical response against *L*. *braziliensis* [23].

In an adaptive clinical trial design it is possible to evaluate the desired outcomes exposing fewer patients without losing the quality of the evidence [38]. The sequential groups with scaled doses allow timely suspension of the research after initial failures with a smaller dose, minimizing unnecessary drug exposure.

During the execution of the study, the first clinical trial evaluating fluconazole in ATL caused by *L*. *braziliensis* was published. The intention-to-treat analysis two months after treatment showed cure rates of 22.2% (6 out of 27) in the fluconazole group and 53.8% (14 out of 26) in the Glucantime group. The per protocol results were the same at six months after the end of treatment. The AEs were similar in both groups [39].

In this study, the cure observed with 450 mg of fluconazole daily against *L*. *guyanensis* was 5% (1 out of 20). Five patients asked to stop taking fluconazole and to receive the rescue therapy. One patient who had clinical failure refused to receive any parenteral medication, and after 120 days he presented no signs of the disease. It is not clear if this outcome was a spontaneous evolution to a cure or a delayed response to the fluconazole treatment.

The remaining 11 patients presented a peculiar clinical outcome. The ulcers, after a period of ten days, began to show remarkable inflammatory signs associated with intense pain, increased size and, in some cases the lymphatic spread of the disease (Fig 3). The early enlargement of the ulcers during treatment with meglumine antimoniate was previously reported, but the intensity of inflammatory signs and the concomitant lymphatic involvement constitutes a novel finding never reported in patients with leishmaniasis exposed to fluconazole [40].

The mechanism of this inflammatory phenomenon deserves more investigation, considering that this high failure rate may be justified by an unrecognized immunological mediated effect associated to fluconazole.

All 16 patients that received Glucantime as rescue therapy were cured after 30 days. One patient moved from Manaus and the researchers lost contact.

Fluconazole was well tolerated systemically, with five cases of mild AEs.

In conclusion, fluconazole, at the dose of 450mg per day, is not efficacious against leishmaniasis in adult men infected by *L*. *guyanensis*. The clinical worsening during fluconazol exposure deserves more attention and should be evaluated in future studies involving fluconazole or other azole therapies.

## Supporting information

S1 Trend Checklist (PDF)

S1 Table Clinical trials treating cutaneous leishmaniasis caused by *Leishmania guyanensis*. (DOCX)

S1 Fig This picture shows the ulcer on patient 1 before treatment and the same patient after 60 days without any evidence of the disease. (TIF)

S1 References Supporting information references in the S1 Table. (DOCX)
