# Supplementary material for: Failure of fluconazole in treating cutaneous leishmaniasis caused by Leishmania guyanensis in the Brazilian Amazon: An open, nonrandomized phase 2 trial
Source: PLoS Negl Trop Dis. 2018 Feb 26;12(2):e0006225. doi: 10.1371/journal.pntd.0006225 (PMC5854414; doi:10.1371/journal.pntd.0006225)
Supplement: S1 Table — (DOCX) [file pntd.0006225.s002.docx]

S1 Table.Clinical trials treating cutaneous leishmaniasis caused by *Leishmania guyanensis.*

| Reference | Cure Rate | | | Country |
| --- | --- | --- | --- | --- |
|  | Glucantime | Pentamidine | Miltefosine |  |
| Romero et al., 2001 [14] | 30,6%  (15/49) | - | - | Brazil |
| Nacher et al.,  2001[41] | - | 87%  (172/198) | - | French Guiana |
| Arevalo et al.,  2007 [42] | 91.7%  (22/24) | - | - | Peru |
| Van der Meide et al.,  2009 [43] | - | 78%  (17/23) | - | Suriname |
| Chrusciak-Talhari et al.,  2011 [15] | 53.6%  (15/28) | - | 71.4%  (40/56) | Brazil |
| Neves et al.,  2011 [13] | 53.8%  (35/58) | 57.1%  (36/58) | - | Brazil |
| Rubiano et al., 2012 [16] | 80%  (4/5) | - | 54.5%  (6/11) | Colombia |
| Mans et al.,  2015 [41] | - | 74.1%  (66/89) | - | French Guiana |
