# Supplementary figures and images for: Failure of fluconazole in treating cutaneous leishmaniasis caused by Leishmania guyanensis in the Brazilian Amazon: An open, nonrandomized phase 2 trial
Source: PLoS Negl Trop Dis. 2018 Feb 26;12(2):e0006225. doi: 10.1371/journal.pntd.0006225 (PMC5854414; doi:10.1371/journal.pntd.0006225)

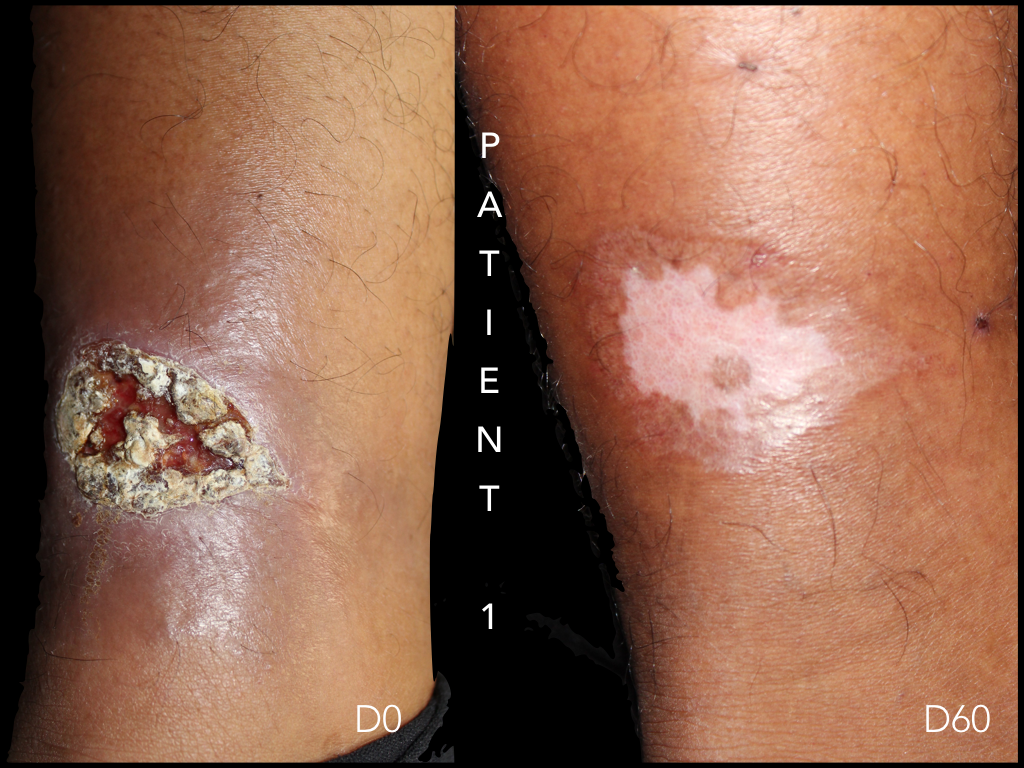

Supplement: S1 Fig — (TIF) [file pntd.0006225.s003.tif]
