# Supplementary material for: Failure of fluconazole in treating cutaneous leishmaniasis caused by Leishmania guyanensis in the Brazilian Amazon: An open, nonrandomized phase 2 trial
Source: PLoS Negl Trop Dis. 2018 Feb 26;12(2):e0006225. doi: 10.1371/journal.pntd.0006225 (PMC5854414; doi:10.1371/journal.pntd.0006225)
Supplement: S1 References — (DOCX) [file pntd.0006225.s004.docx]

S1 References.

Supporting Information References in the S1 Table

13. Neves LO, Talhari AC, Gadelha EP, Silva Junior RM, Guerra JA, Ferreira LC, et al. A randomized clinical trial comparing meglumine antimoniate, pentamidine and amphotericin B for the treatment of cutaneous leishmaniasis by *Leishmania guyanensis*. Anais brasileiros de dermatologia. 2011;86(6):1092-101. PubMed PMID: 22281895.

14. Romero GA, Guerra MV, Paes MG, Macedo VO. Comparison of cutaneous leishmaniasis due to *Leishmania (Viannia) braziliensis* and *L. (V.) guyanensis* in Brazil: therapeutic response to meglumine antimoniate. The American Journal of Tropical Medicine and Hygiene. 2001;65(5):456-65. PubMed PMID: 11716098.

15. Chrusciak-Talhari A, Dietze R, Chrusciak Talhari C, da Silva RM, Gadelha Yamashita EP, de Oliveira Penna G, et al. Randomized controlled clinical trial to access efficacy and safety of miltefosine in the treatment of cutaneous leishmaniasis caused by *Leishmania (Viannia) guyanensis* in Manaus, Brazil. The American Journal of Tropical Medicine and Hygiene. 2011;84(2):255-60. doi: 10.4269/ajtmh.2011.10-0155. PubMed PMID: 21292895; PubMed Central PMCID: PMC3029178.

16. Rubiano LC, Miranda MC, Muvdi Arenas S, Montero LM, Rodriguez-Barraquer I, Garcerant D, et al. Noninferiority of miltefosine versus meglumine antimoniate for cutaneous leishmaniasis in children. The Journal of Infectious Diseases. 2012;205(4):684-92. doi: 10.1093/infdis/jir816. PubMed PMID: 22238470; PubMed Central PMCID: PMC326613641. **Nacher** M, Carme B, Sainte Marie D, Couppié P, Clyti E, Guibert P, Pradinaud R. [Influence of clinical presentation on the efficacy of a short course of pentamidine in the treatment of cutaneous leishmaniasis in French Guiana.](https://www.ncbi.nlm.nih.gov/pubmed/11454242) Ann Trop Med Parasitol. 2001 Jun;95(4):331-6. PMID:11454242.

42. [Arevalo J](https://www.ncbi.nlm.nih.gov/pubmed/?term=Arevalo%20J%5BAuthor%5D&cauthor=true&cauthor_uid=17492601)^1^, [Ramirez L](https://www.ncbi.nlm.nih.gov/pubmed/?term=Ramirez%20L%5BAuthor%5D&cauthor=true&cauthor_uid=17492601), [Adaui V](https://www.ncbi.nlm.nih.gov/pubmed/?term=Adaui%20V%5BAuthor%5D&cauthor=true&cauthor_uid=17492601), [Zimic M](https://www.ncbi.nlm.nih.gov/pubmed/?term=Zimic%20M%5BAuthor%5D&cauthor=true&cauthor_uid=17492601), [Tulliano G](https://www.ncbi.nlm.nih.gov/pubmed/?term=Tulliano%20G%5BAuthor%5D&cauthor=true&cauthor_uid=17492601), [Miranda-Verástegui C](https://www.ncbi.nlm.nih.gov/pubmed/?term=Miranda-Ver%C3%A1stegui%20C%5BAuthor%5D&cauthor=true&cauthor_uid=17492601), [Lazo M](https://www.ncbi.nlm.nih.gov/pubmed/?term=Lazo%20M%5BAuthor%5D&cauthor=true&cauthor_uid=17492601), [Loayza-Muro R](https://www.ncbi.nlm.nih.gov/pubmed/?term=Loayza-Muro%20R%5BAuthor%5D&cauthor=true&cauthor_uid=17492601), [De Doncker S](https://www.ncbi.nlm.nih.gov/pubmed/?term=De%20Doncker%20S%5BAuthor%5D&cauthor=true&cauthor_uid=17492601), [Maurer A](https://www.ncbi.nlm.nih.gov/pubmed/?term=Maurer%20A%5BAuthor%5D&cauthor=true&cauthor_uid=17492601), [Chappuis F](https://www.ncbi.nlm.nih.gov/pubmed/?term=Chappuis%20F%5BAuthor%5D&cauthor=true&cauthor_uid=17492601), [Dujardin JC](https://www.ncbi.nlm.nih.gov/pubmed/?term=Dujardin%20JC%5BAuthor%5D&cauthor=true&cauthor_uid=17492601), [Llanos-Cuentas A](https://www.ncbi.nlm.nih.gov/pubmed/?term=Llanos-Cuentas%20A%5BAuthor%5D&cauthor=true&cauthor_uid=17492601). **Influence of *Leishmania (Viannia)* species on the response to antimonial treatment in patients with American tegumentary leishmaniasis.** [Journal of Infectious Diseases.](https://www.ncbi.nlm.nih.gov/pubmed/?term=Arevalo+2007+guyanensis) 2007 15;195(12):1846-51. doi: 10.1086/518041. PMID: 17492601

43. [van der Meide WF](https://www.ncbi.nlm.nih.gov/pubmed/?term=van%20der%20Meide%20WF%5BAuthor%5D&cauthor=true&cauthor_uid=19126051), [Sabajo LO](https://www.ncbi.nlm.nih.gov/pubmed/?term=Sabajo%20LO%5BAuthor%5D&cauthor=true&cauthor_uid=19126051), [Jensema AJ](https://www.ncbi.nlm.nih.gov/pubmed/?term=Jensema%20AJ%5BAuthor%5D&cauthor=true&cauthor_uid=19126051), [Peekel I](https://www.ncbi.nlm.nih.gov/pubmed/?term=Peekel%20I%5BAuthor%5D&cauthor=true&cauthor_uid=19126051), [Faber WR](https://www.ncbi.nlm.nih.gov/pubmed/?term=Faber%20WR%5BAuthor%5D&cauthor=true&cauthor_uid=19126051), [Schallig HD](https://www.ncbi.nlm.nih.gov/pubmed/?term=Schallig%20HD%5BAuthor%5D&cauthor=true&cauthor_uid=19126051), [Fat RF](https://www.ncbi.nlm.nih.gov/pubmed/?term=Fat%20RF%5BAuthor%5D&cauthor=true&cauthor_uid=19126051). Evaluation of treatment with pentamidine for cutaneous leishmaniasis in Suriname. [International Journal of Dermatology.](https://www.ncbi.nlm.nih.gov/pubmed/?term=Meide+2009+guyanensis) 2009 Jan;48(1):52-8.

doi: 10.1111/j.1365-4632.2009.03883.x. PMID:19126051
